# Delayed administration of nafamostat mesylate inhibits thrombin-mediated blood–spinal cord barrier breakdown during acute spinal cord injury in rats

**DOI:** 10.1186/s12974-022-02531-w

**Published:** 2022-07-16

**Authors:** Chenxi Zhao, Tiangang Zhou, Xiaoqing Zhao, Yilin Pang, Wenxiang Li, Baoyou Fan, Ming Li, Xinjie Liu, Lei Ma, Jiawei Zhang, Chao Sun, Wenyuan Shen, Xiaohong Kong, Xue Yao, Shiqing Feng

**Affiliations:** 1 Department of Orthopedics, International Science and Technology Cooperation Base of Spinal Cord Injury, Tianjin Key Laboratory of Spine and Spinal Cord Injury, Tianjin Medical University General Hospital, 154 Anshan Road, Heping District, Tianjin, China; 2 Department of Orthopedics, Orthopedic Research Center of Shandong University, Cheeloo College of Medicine, Qilu Hospital of Shandong University, Jinan, Shandong China

**Keywords:** Nafamostat mesylate, Thrombin, Administration time window, Spinal cord injury, Blood–spinal cord barrier, Neuroinflammation

## Abstract

**Background:**

Nafamostat mesylate (nafamostat, NM) is an FDA-approved serine protease inhibitor that exerts anti-neuroinflammation and neuroprotective effects following rat spinal cord injury (SCI). However, clinical translation of nafamostat has been limited by an unclear administration time window and mechanism of action.

**Methods:**

Time to first dose of nafamostat administration was tested on rats after contusive SCI. The optimal time window of nafamostat was screened by evaluating hindlimb locomotion and electrophysiology. As nafamostat is a serine protease inhibitor known to target thrombin, we used argatroban (Arg), a thrombin-specific inhibitor, as a positive control in the time window experiments. Western blot and immunofluorescence of thrombin expression level and its enzymatic activity were assayed at different time points, as well its receptor, the protease activated receptor 1 (PAR1) and downstream protein matrix metalloproteinase-9 (MMP9). Blood–spinal cord barrier (BSCB) permeability leakage indicator Evans Blue and fibrinogen were analyzed along these time points. The infiltration of peripheral inflammatory cell was observed by immunofluorescence.

**Results:**

The optimal administration time window of nafamostat was 2–12 h post-injury. Argatroban, the thrombin-specific inhibitor, had a similar pattern. Thrombin expression peaked at 12 h and returned to normal level at 7 days post-SCI. PAR1, the thrombin receptor, and MMP9 were significantly upregulated after SCI. The most significant increase of thrombin expression was detected in vascular endothelial cells (ECs). Nafamostat and argatroban significantly downregulated thrombin and MMP9 expression as well as thrombin activity in the spinal cord. Nafamostat inhibited thrombin enrichment in endothelial cells. Nafamostat administration at 2–12 h after SCI inhibited the leakage of Evans Blue in the epicenter and upregulated tight junction proteins (TJPs) expression. Nafamostat administration 8 h post-SCI effectively inhibited the infiltration of peripheral macrophages and neutrophils to the injury site.

**Conclusions:**

Our study provides preclinical information of nafamostat about the administration time window of 2–12 h post-injury in contusive SCI. We revealed that nafamostat functions through inhibiting the thrombin-mediated BSCB breakdown and subsequent peripheral immune cells infiltration.

**Supplementary Information:**

The online version contains supplementary material available at 10.1186/s12974-022-02531-w.

## Background

Traumatic spinal cord injury is a severe central nervous system (CNS) disease with no effective treatment [1, 2]. At present, the best clinical medication for spinal cord injury (SCI) is methylprednisolone (MP), which is mildly beneficial by inhibiting the acute inflammatory cascade [3]. However, its narrow administration time window (< 8 h post-injury, hpi) and side effect limit its clinical application. Although a few neuroprotective compounds have been identified in animal SCI models [4], their clinical transformation is often hindered by their safety, impractical administration time window and unclear mechanism.

Nafamostat mesylate is a synthetic spectrum serine protease inhibitor [5] that has been widely used in patients with pancreatitis and disseminated intravascular coagulopathy and therefore has a known administration safety profile. In recent years, nafamostat has been shown to have neuroprotective and anti-inflammatory effects on CNS diseases [6, 7]. Nafamostat can also improve neurological outcome and axonal regeneration after stroke and protect against acute cerebral ischemia via preserving the blood–brain barrier (BBB) [8]. In our previous study, nafamostat inhibited neuroinflammation during acute phase of injury and promoted locomotor function recovery post-SCI [9]. However, its administration time window and repair mechanism in SCI remains elusive.

Mosaic serine proteases have diversified effects on SCI. There is protective effect on increasing neuron and glial cell proliferation and migration by tissue-type plasminogen activator (tPA) [8, 10], as well as damaging effect on apoptosis and activating inflammatory and immune response by thrombin [11]. Serine protease neuropsin and protease M/neuros expressed by oligodendrocyte upregulate after SCI, and promote demyelination [12]. Thrombin, a widely studied serine protease targeted by nafamostat, is a multifunctional enzyme that is restricted from the CNS under physiological conditions [11]. Recent research has shown that it has a crucial role in traumatic brain injury (TBI), SCI, neurodegenerative diseases (Alzheimer's and Parkinson's diseases) and ischemic stroke [13, 14]. Furthermore, thrombin can promote astrocyte activation-induced glial scar hyperplasia, neuronal apoptosis and neuropathic pain through protease activated receptor 1 (PAR1) [15–17]. Meanwhile, Machida et al. reported that thrombin produces MMP9 by activating the downstream PAR1 pathway [18]. MMP9 (matrix metalloproteinase 9) participated in the degradation of extravascular matrix and tight junction proteins (TJPs), including Claudin-5, Occludin and zonula occludens-1 (ZO-1) [19], and played an important role in BBB dysfunction. However, the temporal and spatial distribution of thrombin after SCI remains unclear, and such knowledge would benefit the development of the thrombin-targeted drugs for SCI.

As a highly selective, permeable vascular endothelial structure, blood–spinal cord barrier (BSCB) provides strong support for the homeostasis of the spinal cord microenvironment [19, 20]. The impairment of the BSCB aggravates the pathological process of SCI, allowing various molecules and blood cells to enter the spinal cord [21]. In addition to the leakage of blood-derived leukocyte and monocytes infiltration [22], various types of proteases, such as thrombin, plasmin and kallikrein crossed the compromised BSCB, resulting in a perpetual neurological deficiency in acute SCI [9, 17, 24]. Hence, early drug administration targeting the BSCB protection could be beneficial by inhibiting the neuroinflammatory cascade and improving long-term neurological outcomes.

In this paper, we explore the optimal administration time window of nafamostat in SCI, and demonstrate the underlying mechanism. We found that nafamostat promotes the BSCB protection and improves neurological function of rats with SCI by inhibiting thrombin and its downstream signaling pathway.

## Methods

### Animals

Female Wistar rats (8 weeks old, 200–220 g) were purchased from Vital River Laboratory Animal Technology Co., Ltd (Beijing, China). Animals were kept in an environment with a 12/12-h light–dark cycle (light on between 7:00 and 19:00) with a temperature of 20–25 °C and humidity of 40–60%. Standard laboratory food and tap water were given ad libitum. All experimental procedures involving rats were approved by the Ethics Committee of Tianjin Medical University (Tianjin, China, IRM-DWLL-2020080). The animal experimental protocols were performed according to the Animal Research: Reporting of In Vivo Experiments (ARRIVE) guidelines (http://arriveguidelines.org/).

### Spinal cord injury

All surgeries were conducted under aseptic conditions. After deep anesthetization with 4% inhaled isoflurane (R510-22, RWD, Shenzhen, China), the skin on the dorsal side of the spine was shaved and disinfected before surgery. A 1-cm midline longitudinal incision was made centered on T10 through the skin and muscle covering the T10 vertebrae. The spinal cord was exposed by a T10 vertebrae dorsal laminectomy. The NYU Impactor Model III (W.M.Keck, USA) was used to establish the moderate SCI contusion model by a 10 g rod with a height of 25 mm. The muscles and skin were sutured layer by layer. Sham group animals received a T10 laminectomy only. Manual bladder emptying was performed twice a day until the rats were able to urinate by themselves. Antibiotics (Gentamycin sulfate, Solarbio, G8170, Beijing, China) were administrated twice a day in the first 3 days post-injury (dpi).

### Drug administration

Nafamostat mesylate (S1386) and argatroban (S2069), a thrombin-specific inhibitor, were purchased from Selleck (MA, USA). To explore the administration time window for nafamostat and argatroban, we used different first administration times (FAT), including 0 h, 2 h, 8 h, 12 h, 24 h, 48 h, 5 days after SCI. Rats were randomly divided into groups of different FATs. Nafamostat (1 mg/mL, dissolved in 0.9% saline, 10 mg/kg/day) [9] and argatroban (0.3 mg/mL, dissolved in 0.9% saline, 3 mg/kg/day) [24] was administered by intraperitoneal (i.p.) injection at different FATs and then twice a day thereafter until 3 dpi or the endpoints of experiments. The rats in the Sham and SCI groups were injected with the same volume saline.

### Basso, Beattie, and Bresnahan (BBB) locomotor test

The Basso, Beattie, and Bresnahan (BBB) locomotor scores were used to evaluate hindlimb motor function [25]. BBB was performed 1 day before and after surgery and then weekly post-injury. Briefly, the BBB test score of 0 represented no observable movement. The score of 1–7 was used to evaluate the hind joint motion in the early stage of SCI. The score of 8–13 was used to evaluate the gait and coordinated movement of the recovery in the middle stage of SCI, and the score of 14–20 was used to assess the accurate paw movement. The score 21 represented normal movement (Table 1). Before scoring, rats were allowed to roam freely in an open field for 5 min. Each rat was observed at least 2 min and scored independently by at least 3 well-trained investigators. All the investigators were double-blinded.

**Table 1:** BBB Locomotor Scale [25]

Score	Description
0	No observable hindlimb movement
1	Slight movement of one or two joints, usually the hip and/or knee
2	Extensive movement of one joint or extensive movement of one joint and slight movement of one other joint
3	Extensive movement of two joints
4	Slight movement of all three joints of the hindlimb
5	Slight movement of two joints and extensive movement of the third
6	Extensive movement of two joints and slight movement of the third
7	Extensive movement of all three joints of the hindlimb
8	Sweeping with no weight support or plantar placement of the paw with no weight support
9	Plantar placement of the paw with weight support in stance only (i.e., when stationary) or occasional, frequent, or consistent weight supported dorsal stepping and no plantar stepping
10	Occasional weight supported plantar steps, no forelimb–hindlimb coordination
11	Frequent to consistent weight supported plantar steps and no forelimb–hindlimb coordination
12	Frequent to consistent weight supported plantar steps and occasional forelimb–hindlimb coordination
13	Frequent to consistent weight supported plantar steps and frequent forelimb–hindlimb coordination
14	Consistent weight supported plantar steps, consistent forelimb–hindlimb coordination; and predominant paw position during locomotion is rotated (internally or externally) when it makes initial contact with the surface as well as just before it is lifted off at the end of stance or frequent plantar stepping, consistent forelimb–hindlimb coordination, and occasional dorsal stepping
15	Consistent plantar stepping and consistent forelimb–hindlimb coordination; and no toe clearance or occasional toe clearance during forward limb advancement; predominant paw position is parallel to the body at initial contact
16	Consistent plantar stepping and consistent forelimb–hindlimb coordination during gait; and toe clearance occurs frequently during forward limb advancement; predominant paw position is parallel at initial contact and rotated at liftoff
17	Consistent plantar stepping and consistent forelimb–hindlimb coordination during gait; and toe clearance occurs frequently during forward limb advancement; predominant paw position is parallel at initial contact and liftoff
18	Consistent plantar stepping and consistent forelimb–hindlimb coordination during gait; and toe clearance occurs consistently during forward limb advancement; predominant paw position is parallel at initial contact and rotated at liftoff
19	Consistent plantar stepping and consistent forelimb–hindlimb coordination during gait; and toe clearance occurs consistently during forward limb advancement; predominant paw position is parallel at initial contact and liftoff; and tail is down part or all of the time
20	Consistent plantar stepping and consistent coordinated gait; consistent toe clearance; predominant paw position is parallel at initial contact and liftoff; tail consistently up; and trunk instability
21	Consistent plantar stepping and coordinated gait, consistent toe clearance, predominant paw position is parallel throughout stance, consistent trunk stability, tail consistently up

### CatWalk-assisted gait analysis

The locomotion recovery of rats in Sham, SCI, and nafamostat (NM), argatroban (Arg) groups were also tested using the Noldus CatWalk XT system (version 10.6, Noldus, The Netherlands) following the manufacturer’s instructions (CatWalk XT 10.6 Reference Manual) and previous description [26]. Briefly, rats were trained to cross the walkway in the week before testing every day. Gait analysis was performed 5 weeks after SCI in a dark and quiet environment. Parameters including regularity index (RI), step sequence parameters, paw statistics, general parameters (mean speed and cadence), and bases of support were analyzed with CatWalk software version 10.6 by an observer blinded to the treatment.

### Electrophysiological tests

Electrophysiological tests were used to assess nerve conduction in rats 5 weeks post-injury as previously described [27]. Sensory evoked potential (SEP) and motor evoked potential (MEP) of rats were processed by electrophysiological devices (YRKJ-G2008; Zhuhai Yiruikeji Co, Ltd, Guangdong, China). The recovery of sensory and motor nerve function in rats was evaluated according to the detection results. After the rats were deeply anesthetized and skin prepared, a 5.1 Hz square wave of 0.1 ms was generated by a constant stimulator (2 mA) in duration to stimulate the median nerve along the hindlimb for SEP and a single stimulator of 5 mA was used to stimulate the motor area of the cerebral cortex for MEP. For SEP, a total of 50 responses of each rat was averaged.

### Coagulation function detection

The effect of nafamostat intervention on peripheral blood coagulation function of rats at different time points after injury was evaluated to determine medication safety. Three rats were randomly selected from each group of rats administered at different time points, and 1 mL peripheral blood was collected from the epicanthal vein 2 h after the nafamostat intervention. After the blood samples were centrifuged (3000 rpm, 10 min), an automatic coagulation analyzer (RAC030, Kolda, Wuhan, China) was used to detect the prothrombin time (PT), prothrombin activity (PTA) and international normalized ratio (INR).

### Blood–spinal cord barrier permeability test

The permeability of BSCB was investigated with Evans Blue (EB) according to a previous report [28]. Briefly, EB dye (E8010, 10 mg/mL diluted in saline, 1 mL/kg, Solarbio, Beijing, China) was administered through the tail vein and allowed to circulate for 3 h. The spinal cord was resected out after deep anesthesia. The spinal cord was photographed from topical and coronal views to show EB leakage. The injured epicenter of spinal cord was sliced into 8 µm sections under light protection. EB leakage was detected using a Leica fluorescence microscopy (Leica DMi8, Germany) under the excitation of 550-nm wavelength green light. The images were stitched together by Adobe Photoshop CC 2019. The total fluorescence intensity was measured by ImageJ (Version 1.46r; National Institutes of Health, Bethesda, MD, USA).

### Thrombin activity assay

Thrombin activity was measured as previously described [29]. In brief, after perfusion with 4 ℃ PBS, the injured spinal cord epicenter was isolated and cut into 0.5 cm (20–30 mg). Epicenters were homogenized by assay buffer without the substrate (50 mM Tris·HCl, pH 5.8, 150 mM NaCl, 1 mM CaCl_2_, 0.1% BSA). The homogenate was centrifuged at 12000*g* for 13 min at room temperature. Each 50 μL sample was mixed with 150 μL assay buffer mixed with the substrate (protease inhibitor prolyl endopeptidase inhibitor II (20 mM), Benzoyl-Phe-Val-Arg-AMC·HCl (13 mM)). The mixture was added to a 96-well black microplate. The kinetic fluorescence was determined for 45 min at 27 ℃ by a fluorescence detection system (SYNERGY, BioTek; excitation 360/40 nm, emission 450/40 nm). Thrombin substrate Benzoyl-Phe-Val-Arg-AMC·HCl (B7632) and Prolyl Endopeptidase Inhibitor II (537011) were purchased from Merck (Germany).

### Immunofluorescence staining

The rats were perfused with 4 ℃ PBS, followed by 4% paraformaldehyde. 0.5 cm spinal cord segment was resected from the level of epicenter and postfixed in 4% paraformaldehyde solution overnight. The samples were dehydrated through increasing concentration of sucrose solution (10%, 20%, 30%). After embedding with Tissue-Tek OCT compound (4583; SAKURA, Torrance, CA, USA), the tissue was frozen in − 80 ℃ or in liquid nitrogen for long-time preservation. Spinal cord tissue in the injury center of 3 rats were selected in each group for sectioning, each tissue was cut and selected 3 slices for immunofluorescence staining, and the posterior horn of the bilateral spinal cord was selected for each slice to take photos. Tissues were sliced into 8 μm. Slices were incubated with the following rat-specific primary antibodies: GFAP (bs-0199R, Bioss, China), NeuN (ab177487, Abcam, USA), Anti-CD31 antibody (ab24590, Abcam, USA), Anti-CD31 (bs-0195R, Bioss,China), ZO-1 (21773-1-AP, Proteintech, China), Claudin5 (35-2500, Invitrogen, USA), CD68 (ab31630, Abcam, USA), Iba1 (10904-1-AP, Proteintech, China), PDGFR2 (3169s, CST, USA), Fibrinogen (ab34269, Abcam, USA). For visualization, fluorescent Alexa Fluor 488 Goat anti-Rabbit (a0423, Beyotime, China) or Cy3-labeled Goat Anti-Mouse (a0521, Beyotime, China) or fluorescent Alexa Fluor 488 Goat anti-Mouse (a0428, Beyotime, China) or Cy3-labeled Goat Anti-Rabbit (a0516, Beyotime, China) secondary antibodies were used for both or single staining at room temperature for 1 h. Cell nuclei was visualized by Vectashield containing DAPI (ab104139, Abcam, USA). A Leica fluorescence microscope (Leica DMi8, Germany) with 10 × , 20 × , 40 × objectives, Leica camera (Leica TL LED, Germany) and imaging software (Leica Application Suite X, Germany) were used to acquire images. Acquired images were quantified by the software of Fiji (Fiji is just ImageJ, National Institutes of Health, USA).

### Western blot

The expression of thrombin and downstream signaling proteins were quantified by western blot. Injured spinal cord epicenters 0.5 cm in size were harvested from 3 individual rats and lysed by homogenization in RIPA Lysis Buffer (P0013B, Beyotime) (100 μL/10 mg spinal cord) with PhosSTOP (04906837001; phosphatase inhibitor) and cOmplete (04693132001; protease inhibitor) from Roche (Mannheim, Germany). The protein concentration of each sample was measured using the bicinchoninic acid assay (BCA) (BL52A, biosharp, China). After SDS-PAGE and PVDF membrane transfer, antibodies were used to probe different proteins. Anti-Thrombin (ab92621, Abcam, USA), anti-PAR1 (GTX64534, Gene Tex, USA), anti-MMP9 (ab38898, Abcam, USA), and anti-GAPDH (sc-32233, Santa Cruz Biotechnology, USA) were used. The blots were visualized using the Immobilon chemiluminescence system (Immobilon Western, Millipore, MA, USA). Image analysis was performed by Fiji. Statistical comparisons of each protein were shown by the standard error and mean (SEM) across at least 3 individual animals.

### Statistical analyses

Statistical analysis were conducted by GraphPad Prism 9.0 (San Diego, CA, USA). Data were presented as mean ± SEM in figures with error bars. *P* values < 0.05 were considered statistically significant. T-test was used to compare two independent groups. One-way ANOVA with Tukey’s or Dunnett’s multiple comparisons post hoc test was used to compare more than two groups, and a two-way ANOVA with Bonferroni’s post hoc test were used if two variables were independent.

## Results

### Nafamostat improved functional recovery at 0–12 h administration time post-SCI

**Fig. 1:**
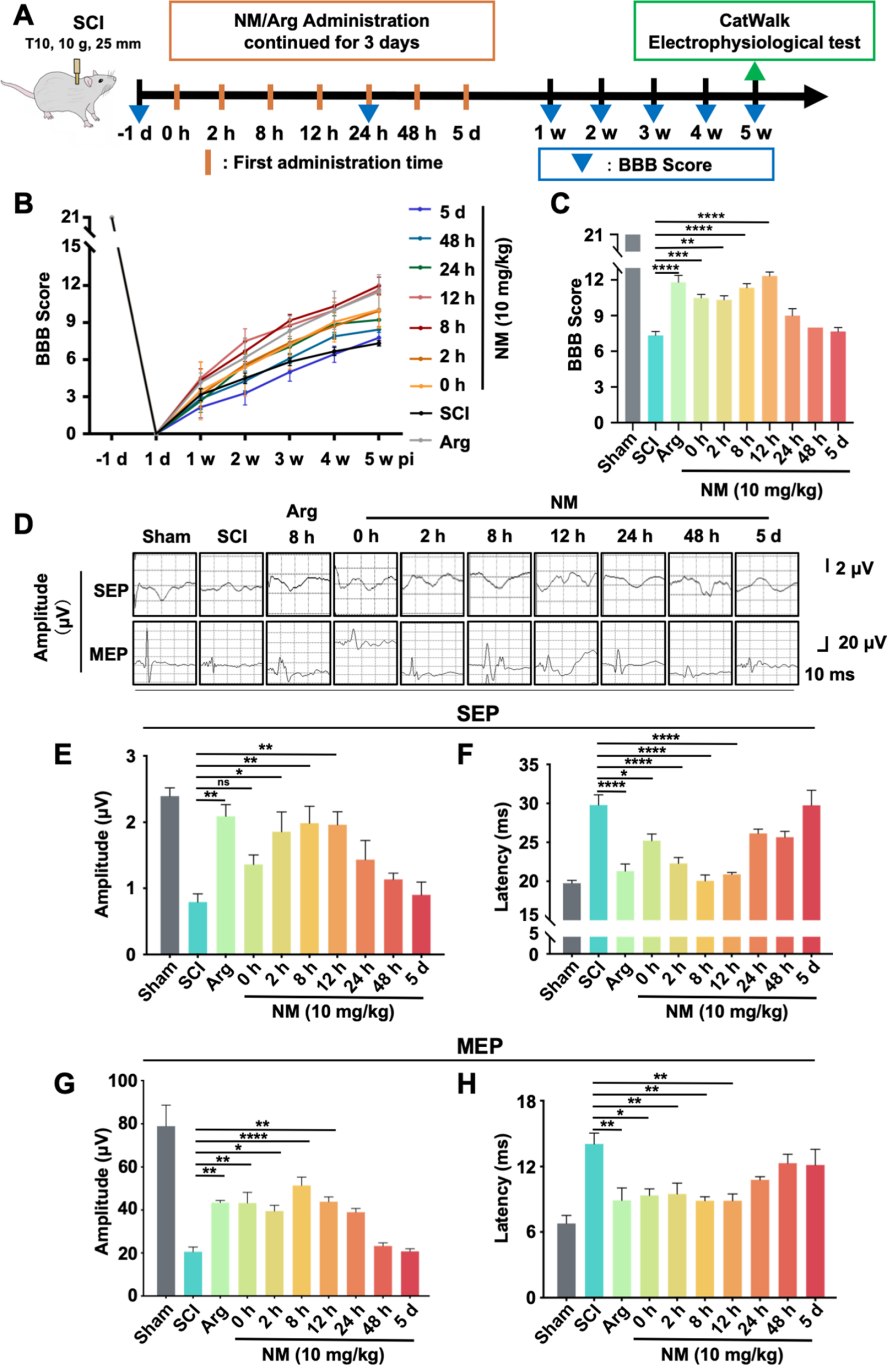
Nafamostat improved functional recovery at 0–12 h administration time after SCI. **A** Illustration describing the experiment design for long-term experiments and motor function recovery. **B** The degree of hindlimb recovery was assessed for 5 w after SCI by BBB score. 7 different FATs of nafamostat (NM) were 0 h, 2 h, 8 h, 12 h, 24 h, 48 h and 5 dpi, and FAT of argatroban (Arg) was 8 hpi (data shown as mean ± SEM, two-way ANOVA with Tukey’s post hoc test, *P < 0.05, **P < 0.01, ***P < 0.001 vs. the SCI group, *n* = 6). **C** Comparison of BBB scores of each group at 5 w point post-injury (data shown as mean ± SEM, one-way ANOVA with Tukey's post hoc test, *P < 0.05, **P < 0.01, ***P < 0.001 vs. the SCI group, *n* = 6). **D** Representative SEP and MEP waveform of nerve electrophysiology examination of rats in each group at 5 w post-SCI. **E**–**H** Quantification of the amplitude and latency of SEP and MEP in each group at 5 w post-SCI (data shown as mean ± SEM, one-way ANOVA with Tukey's post hoc test, *P < 0.05, **P < 0.01, ***P < 0.001 vs. the SCI group, *n* = 6)

**Fig. 2:**
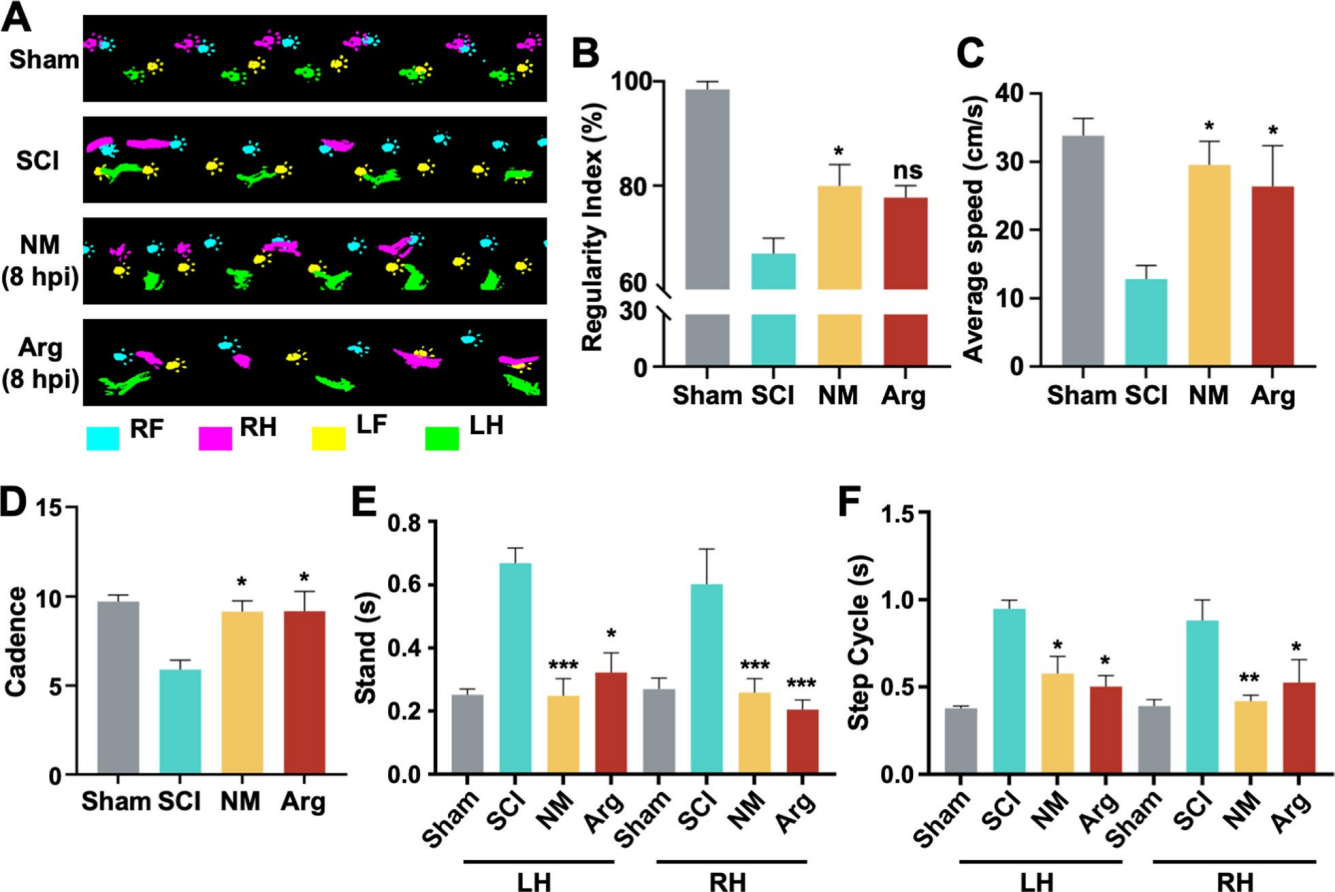
Nafamostat improved the hindlimb motor coordination after SCI. **A** Representative footprints of animals 5 w after injury. **B**–**F** Statistical analysis of regular index (RI) (%), cadence, step cycle (s) of both hindlimbs, stand (s) of both hindlimbs (data shown as mean ± SEM, one-way ANOVA with Tukey's post hoc test, *P < 0.05, **P < 0.01, ***P < 0.001 vs. the SCI group, *n* = 4). *S* Sham, *LF* left forelimb, *RF* right forelimb, *LH* left hindlimb, *RH* right hindlimb

We previously demonstrated that intraperitoneal injection of 10 mg/kg/day nafamostat effectively improves functional recovery after SCI in rats [9], but the optimal administration time was still unclear. To accelerate the clinical transformation of nafamostat, we designed a series of different first administration time (FAT) points to observe functional recovery (Fig. 1A). The FAT time points tested were 0 h, 2 h, 8 h, 12 h, 24 h, 48 h and 5 days post-injury. After the operation, BBB scores were used to quantify locomotor functional recovery in different groups of rats weekly (Fig. 1B). Significantly improved hindlimb function recovery was observed at FATs of 0 h, 2 h, 8 h and 12 h postoperatively. Notably, optimal functional recovery was achieved with 8 h and 12 h FAT (Fig. 1C). At the same time, we also measured the upstream and downstream of nerve conduction function at 5 weeks after SCI in rats by electrophysiology (Fig. 1D). We found that in both SEP and MEP, the amplitude was significantly higher after nafamostat intervention (2 h, 8 h, 12 hpi), especially in the group of 8 h and 12 h (Fig. 1E, G). Meanwhile, the latency period after nafamostat intervention at the time point of 0 h, 2 h, 8 h, 12 h was significantly shorter than that in SCI group (Fig. 1F, H). In conclusion, nafamostat administration at 0–12 h after SCI can effectively improve the recovery of nerve conduction function in rats. Previously, we have shown that argatroban, a selective thrombin synthetic inhibitor, has a therapeutic effect on SCI [30]. Here, we used argatroban as a positive control (Additional file 1: Fig. S1). Results showed that 0–24 h administration of argatroban significantly improved hindlimb function after SCI, as measured using BBB score. The 8-h administration resulted in the best improvement (Additional file 1: Fig. S1A, B). We also found that the amplitude of SEP was increased by the 0–12 h FAT of argatroban (Additional file 1: Fig. S1D), the latency of SEP was shorted by 2–48 h administration (Additional file 1: Fig. S1E). Meanwhile, the amplitude of MEP was increased by the 8–12 h FAT (Additional file 1: Fig. S1F) and the latency of MEP was shorted by 0 h-5 days administration (Additional file 1: Fig. S1G). Therefore, we suggested that argatroban improves the nerve function after SCI with a 2–12 hpi administration time window, and the optimal administration time of argatroban is 8 hpi. Based on these data, we widened the effective time window of nafamostat intervention (0–12 h). To further confirm the optimal time window for nafamostat administration, we performed a gait analysis by the Noldus CatWalk XT system on rats of the 8-h administration group at 5 weeks (Fig. 2). Specifically, both nafamostat (8 hpi) and argatroban (8 hpi) groups led to significant improvement in cadence, step cycle and stand of both hindlimbs. However, only nafamostat significantly improved the regularity index (RI) (%) after SCI significantly. This evidence indicates that the strength and coordination of the hindlimbs of rats after SCI were significantly enhanced by nafamostat administration.

Together, these data demonstrate that 0–12 h after SCI is the effective time window of nafamostat intervention. Nafamostat treatment in this window can notably improve the recovery of motor function, nerve conduction function and hindlimb coordination after SCI, especially at 8 hpi.

### Nafamostat administration after 2 h post-SCI did not affect the coagulation function

Given that inhibition of thrombin may increase the risk of blood clotting dysfunction, we also measured the clotting function using screened FATs that were effective for SCI repair, and selected three parameters closely related to thrombin function for preliminary safety assessment (Additional file 2: Fig. S2). The results of prothrombin time (PT) (Additional file 2: Fig. S2A), prothrombin activity (PTA) (Additional file 2: Fig. S2B), and prothrombin time international normalized ratio (PT-INR) (Additional file 2: Fig. S2C) revealed that the 0 h FAT group had increased clotting dysfunction but the 2–12 h FAT groups revealed no risk of clotting disorders.

Together with behavioral evaluation, we conclude that 2–12 h after SCI is the safe and optimal time window for nafamostat intervention to repair SCI.

### The spatio-temporal pattern of thrombin post-SCI

**Fig. 3:**
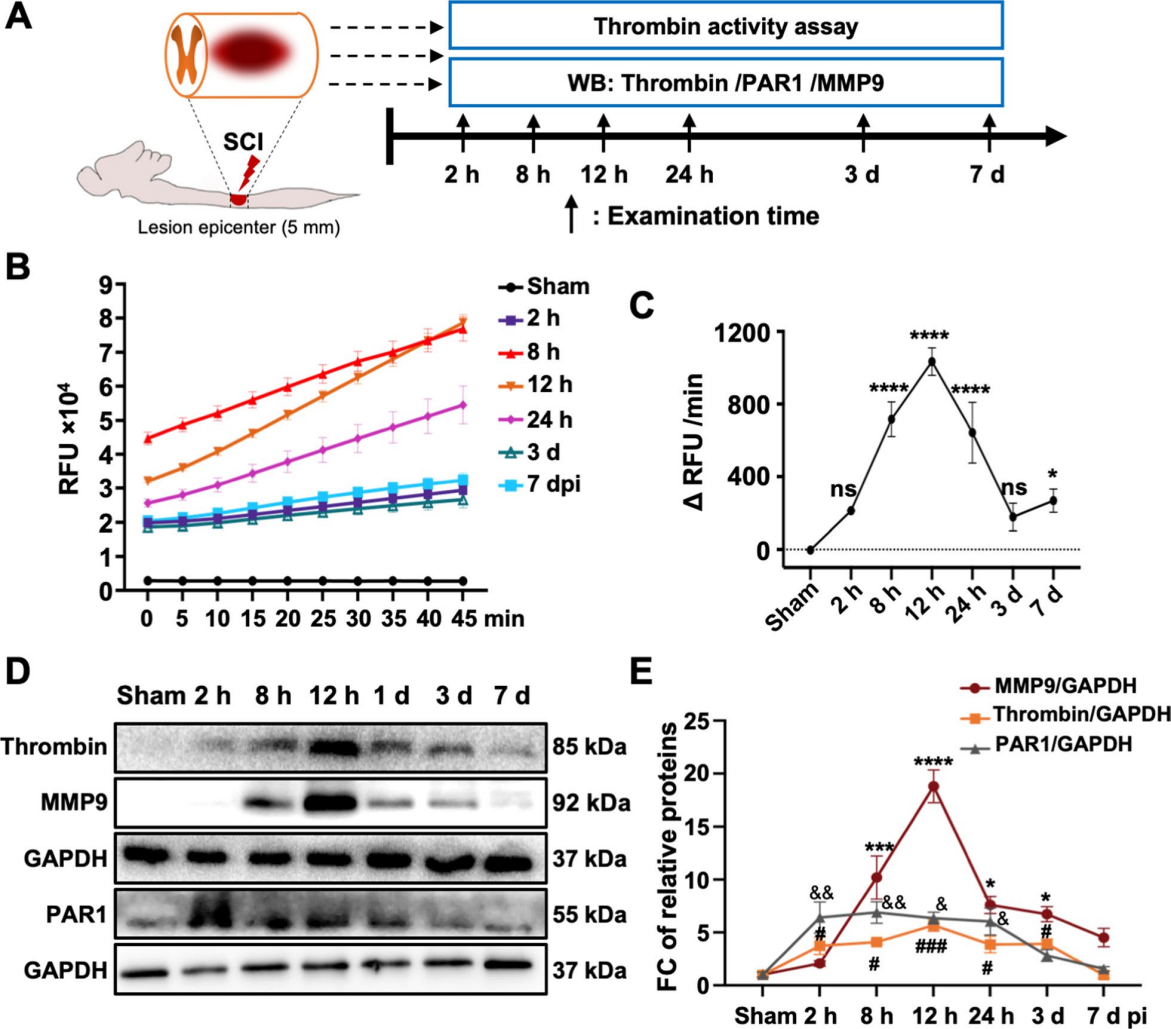
Thrombin activity and expression pattern of thrombin, PAR1 and MMP9 after SCI. **A** Illustration describing the experiment design for temporal expression of thrombin and its downstream proteins. **B** Representative line chart of temporal thrombin activity after SCI (2 h, 8 h, 12 h, 24 h, 3 d, 7 d). **C** Quantitative analysis of thrombin activity (data shown as mean ± SEM, one-way ANOVA with Tukey's post hoc test, *P < 0.05, **P < 0.01, ***P < 0.001, *n* = 3). **D** Representative western blot image of the temporal expression after SCI (2 h, 8 h, 12 h, 24 h, 3 d, 7 d) of thrombin, PAR1 and MMP9. The expression level of each protein was normalized by GAPDH. **E** Quantitative analysis of the temporal expression of MMP9, thrombin and PAR1 normalized by GAPDH (data shown as mean ± SEM, one-way ANOVA with Tukey's post hoc test, *P* value of MMP9 was shown as *, thrombin as #, and PAR1 as &, *P < 0.05, **P < 0.01, ***P < 0.001 vs. the SCI group, *n* = 3)

**Fig. 4:**
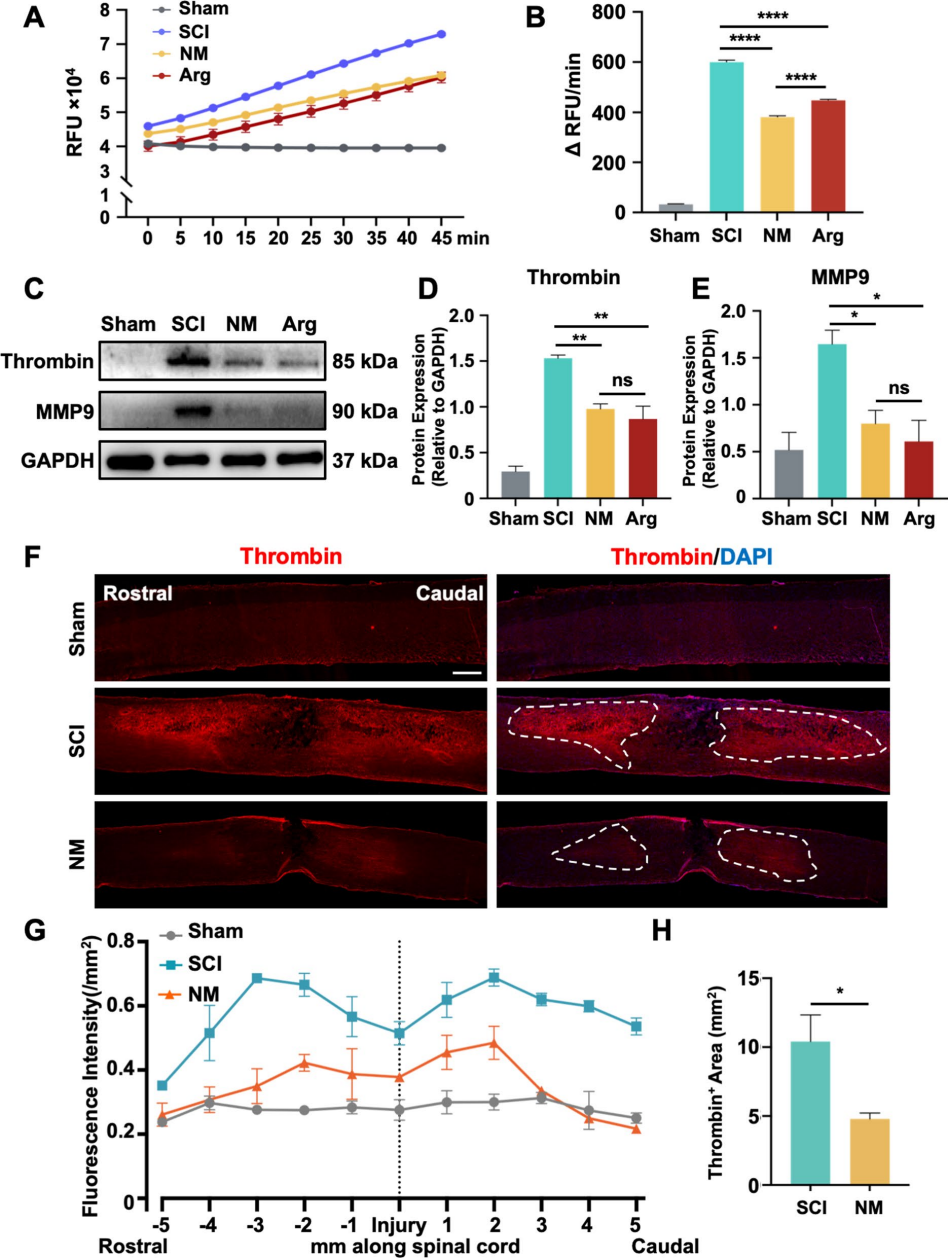
Nafamostat inhibited the expression of thrombin and MMP9 after SCI in the injured spinal cord. **A** Representative line chart of thrombin activity at 3 dpi. **B** Quantitative analysis of thrombin activity (data shown as mean ± SEM, one-way ANOVA with Tukey's post hoc test, ****P < 0.0001, *n* = 5). **C** Representative western blot image of thrombin and MMP expression 3 days after SCI. **D**, **E** Quantitative analysis of thrombin and MMP9 expression level (data shown as mean ± SEM, unpaired t-test, *P < 0.05, **P < 0.01, ***P < 0.001, *n* = 3). **F** Representative longitude immunofluorescence image of thrombin (red) at 3 days post-injury. Cell nuclei were stained with DAPI (blue). Scale bar = 800 μm. The dotted lines represent area where thrombin infiltrates the spinal parenchyma. **G** Quantitative analysis of the fluorescence intensity of thrombin at different locations with the injury site as the center. **H** Quantitative analysis of the size of thrombin infiltration area (data shown as mean ± SEM, unpaired t-test, *P < 0.05, *n* = 3)

Nafamostat is a broad-spectrum serine protease inhibitor whose main target is thrombin [31]. To investigate the mechanism of nafamostat in repairing SCI, we assessed the distribution of thrombin and its downstream proteins post-SCI (Fig. 3A). First, we first examined the activity of thrombin in spinal cord (Fig. 3B, C), finding that the thrombin activity exhibited a time-associated pattern of activity following SCI. The activity of thrombin increased starting at 2 h, peaked at 12 h post-SCI, and maintained a high expression level until 3 dpi. Next, we investigated thrombin and MMP9 protein levels after SCI (Fig. 3D). Like the thrombin activity, at 2 h post-SCI, the protein level of thrombin in the injury epicenter increased about 3.75-fold, peaked at 12 h (5.68-fold) and maintained a high level of expression until 3 dpi (Fig. 3D, E). A significant increase of PAR1 was also observed after SCI during the acute phase. Interestingly, the temporal expression pattern of MMP9 was similar to that of thrombin and PAR1, and the changes of MMP9 were more dramatic than that of thrombin. We observed a twofold upregulation of MMP9 2 hpi that did not reach statistical significance (*P* = 0.07). Gradually, the expression of MMP9 was sharply increased with a tenfold at 8 h and 20-fold at 12 hpi, and still maintained a high level until 3 dpi which was the same with thrombin. Based on the above data, we conclude that the effective time window of nafamostat is completely consistent with the temporal expression of thrombin and MMP9 after SCI. We also detected the thrombin activity at 3 dpi in the spinal cord epicenter (Fig. 4A, B), finding that the thrombin activity was significantly inhibited by nafamostat, which showed a similar inhibitory effect as argatroban. Then we questioned whether nafamostat could inhibit thrombin and MMP9 in the spinal cord parenchyma. We found a sharp decrease of thrombin expression after both nafamostat and argatroban administration at 3 dpi (Fig. 4C, D). And a decrease of MMP9 was found along with the inhibition of thrombin (Fig. 4C, E). We also performed immunofluorescent staining on longitudinal sections at 3 dpi to assess the spatial distribution of thrombin (Fig. 4F). Results showed that thrombin infiltrated into the spinal parenchyma about ± 3 mm around the epicenter after SCI. Nafamostat administration can inhibit both the amount and area of thrombin infiltration (Fig. 4G, H). These results indicated that nafamostat inhibits thrombin activity and protein expression level, with a corresponding decrease in the expression of MMP9.

### Nafamostat inhibited the expression of thrombin, PAR1 and MMP9 in endothelial cells

To further explore the mechanism of nafamostat in SCI repair, we investigated the cell-specific expression of thrombin (Additional file 3: Fig. S3). We found expression of thrombin, which did not alter much after SCI in neurons, astrocytes, microglia and pericytes (Additional file 3: Fig. S3A, C, D, E). Notably, we found that the greatest change of thrombin expression came from CD31^+^ endothelial cells (ECs) compared with the Sham group among the cells in spinal cord (Additional file 3: Fig. S3F). And further, Olig2^+^ oligodendrocyte did not express thrombin before or after SCI (Additional file 3: Fig. S3B). We also analyzed the fibrinogen, as an indicator of BSCB leakage after SCI (Additional file 4: Fig. S4). Fibrinogen only expressed in blood vessels (orange arrow) in Sham group, but it leaked out of blood vessels and infiltrated into spinal cord parenchyma (white arrow) after SCI (Additional file 4: Fig. S4), indicating leakage of BSCB. Together, these results demonstrated the spatio-temporal distribution in the epicenter of the spinal cord after SCI. Thrombin expression and activity peaked at 12 hpi. Thrombin expression was mainly concentrated in vascular ECs after SCI.

**Fig. 5:**
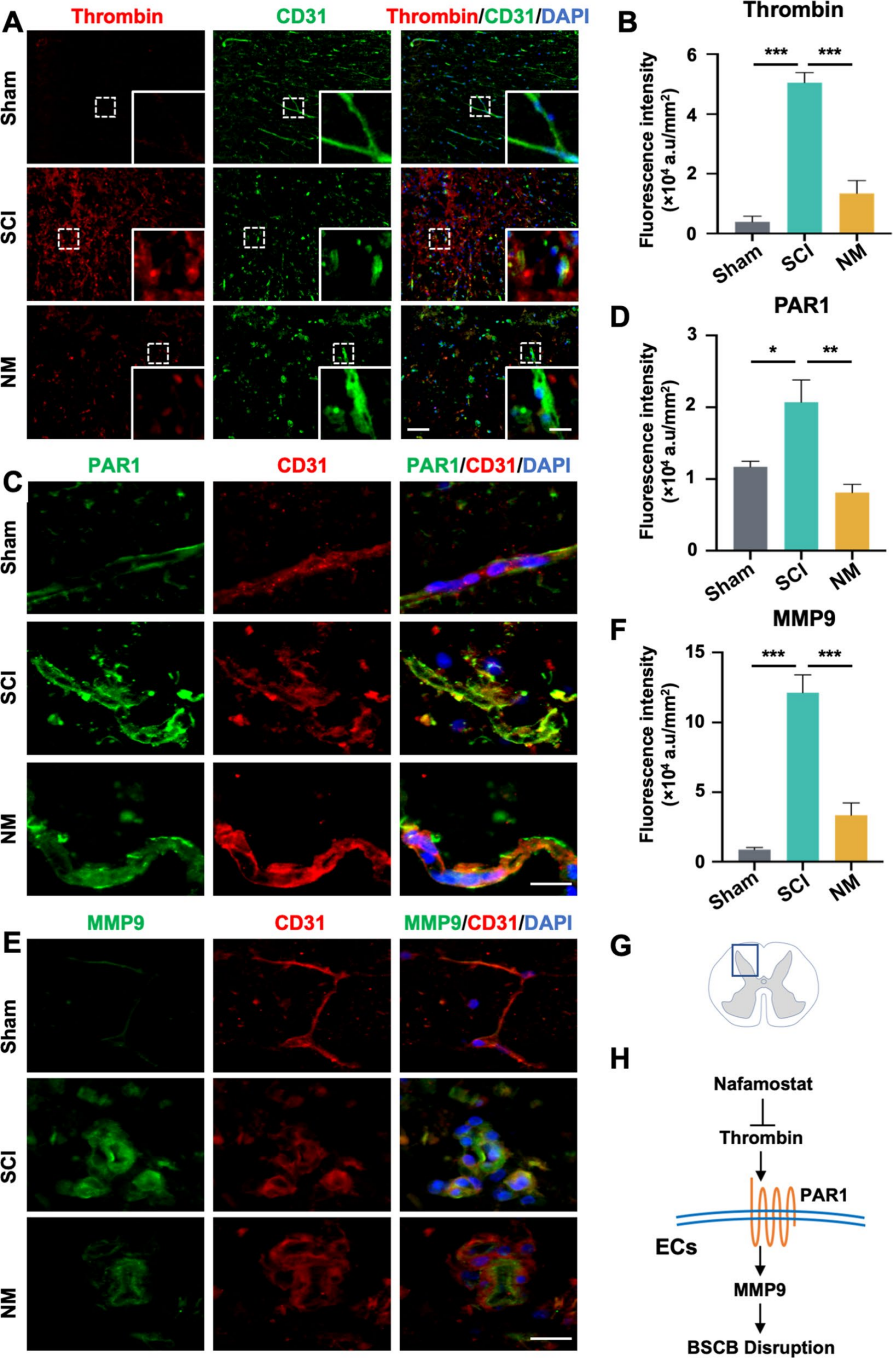
Nafamostat inhibited the expression of thrombin, PAR1 and MMP9 in endothelial cells. **A** Immunofluorescence image of thrombin (red) and CD31 (green) at 3 dpi. Cell nuclei were stained with DAPI (blue). Scale bar = 100 μm, 25 μm. **B** Quantitative analysis of the relative fluorescence intensity of thrombin (data shown as mean ± SEM, one-way ANOVA with Tukey's post hoc test, ***P < 0.001, *n* = 3). **C** Immunofluorescence image of CD31 (red) and PAR1 (green). Cell nuclei were stained with DAPI (blue). Scale bar = 25 μm. **D** Quantitative analysis of the relative fluorescence intensity of PAR1 (data shown as mean ± SEM, one-way ANOVA with Tukey's post hoc test, *P < 0.05, **P < 0.01, *n* = 3). **E** Immunofluorescence image of CD31 (red) and MMP9 (green). Cell nuclei were stained with DAPI (blue). Scale bar = 10 μm. **F** Quantitative analysis of the relative fluorescence intensity of MMP9 (data shown as mean ± SEM, one-way ANOVA with Tukey's post hoc test, ***P < 0.001, *n* = 3). **G** The schematic diagram describes the location of the fluorescent photo. **H** Model diagram of the effect pathway of nafamostat on ECs after SCI

Therefore, we further explored the changes in thrombin expression around vascular ECs after nafamostat intervention. Immunofluorescence results showed that thrombin enrichment in vascular ECs was significantly reduced by nafamostat treatment (Fig. 5A, B). Meanwhile, nafamostat intervention also significantly reduce fibrinogen infiltration (Fig. S4), illustrating that circulation-derived thrombin also reduced. ECs expressed PAR1 abundantly, and PAR1 expression was inhibited by nafamostat intervention, indicating that ECs can be regulated by thrombin (Fig. 5C, D). The upregulated expression of MMP9 after SCI was also concentrated around the ECs, while both the expression of MMP9 and its close relationship with the blood vessels were inhibited after nafamostat intervention (Fig. 5E, F).

Together, nafamostat effectively inhibited the activity and expression of thrombin post-SCI, particularly in vascular ECs. Meanwhile, PAR1 and MMP9 expression were also significantly inhibited (Fig. 5H).

### Nafamostat rescued blood–spinal cord barrier breakdown post-SCI

**Fig. 6:**
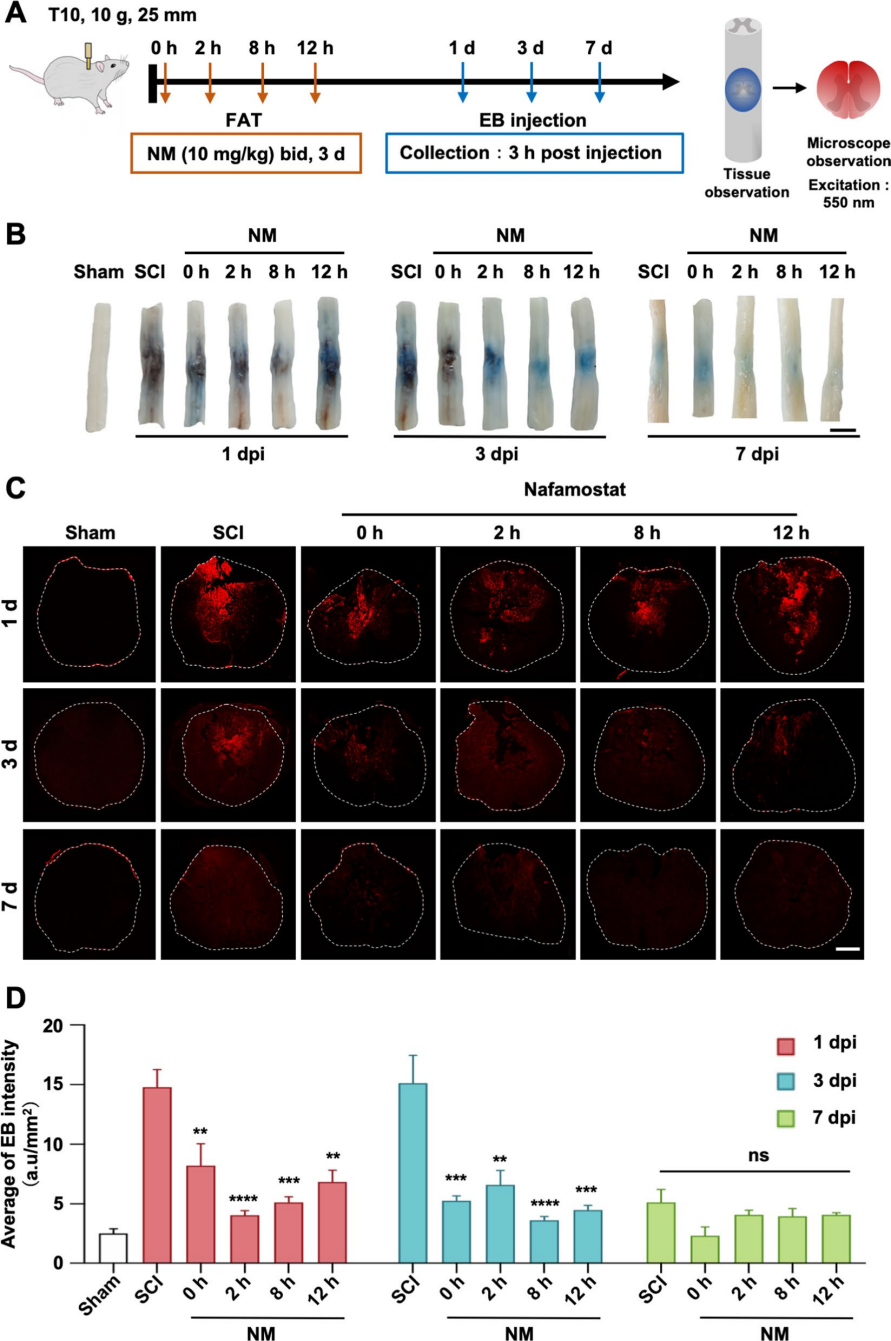
Nafamostat prevented blood–spinal cord barrier breakdown after SCI in rats. **A** Illustration describing the experiment design for detection of the permeability of BSCB by Evans Blue (EB) leakage. After different FAT treatment of NM (10 mg/kg, bid) for 3 days after SCI, 1 mL EB (10 mg/mL) was injected by tail vein. EB solution was allowed to circulate for 3 h before rats were sacrificed. EB leakage was detected using fluorescence microscopy (FM) under the excitation of 550-nm wavelength light. bid: bis in die. **B** Representative photos of general histogram of EB leakage after SCI at 1 d, 3 d, 7 d post-injury of first administration, respectively. Scale bar = 1 cm. **C** Representative fluorescence microscopy photographs of EB (red). Rows represent different groups, and column represents days post-injury. The border of the spinal cord is framed by white dotted line. Scale bar = 500 μm. **D** Quantitative analysis of average fluorescence intensity of EB in spinal cord (data shown as mean ± SEM, one-way ANOVA with Tukey's post hoc test, **P < 0.01, ***P < 0.001 vs. the SCI group, *n* = 4)

We have demonstrated the inhibitory effect of nafamostat on thrombin which results in the reduction of MMP9, especially around ECs. Given that a high level of thrombin and MMP9 in the CNS resulted in a destructive BBB dysfunction[32], we asked whether nafamostat regulated BSCB function by inhibiting thrombin and thus improved the outcome post-SCI. Based on our functional studies (see above), we selected the effective FATs (0 h, 2 h, 8 h and 12 hpi). To determine the integrity of the BSCB, EB (10 mg/mL per rat) was injected via tail vein (Fig. 6A). After 3 h-circulation in the body, the epicenter of the spinal cord was dissected. We saw an obvious change in EB content in the spinal cord parenchyma (Fig. 6B). Furthermore, we quantified the fluorescence intensity of EB in the spinal cord epicenter (Fig. 6C, D). As shown, at 1 dpi and 3 dpi, there was an obvious leakage of EB in the epicenter, suggesting severe damage of BSCB function in the acute SCI. The leakage of EB was attenuated after the intervention of nafamostat. At 1 dpi, 2 h group had the most significant improvement of EB leakage, and there was also significant improvement in the group of 0 h, 8 h and 12 h. While at 3dpi, there was a significant decrease of EB leakage in the 0–12 h administration. Notably, at 7 dpi (the subacute phase), no significant difference was observed between rats with or without nafamostat intervention on EB leakage. Here, we proved that the repair time window of nafamostat on BSCB permeability was completely consistent with the time window of functional recovery of SCI, indicating the mechanism of nafamostat repairing SCI involves its protective effect on BSCB.

### Nafamostat preserved the BSCB tight junction proteins post-SCI

**Fig. 7:**
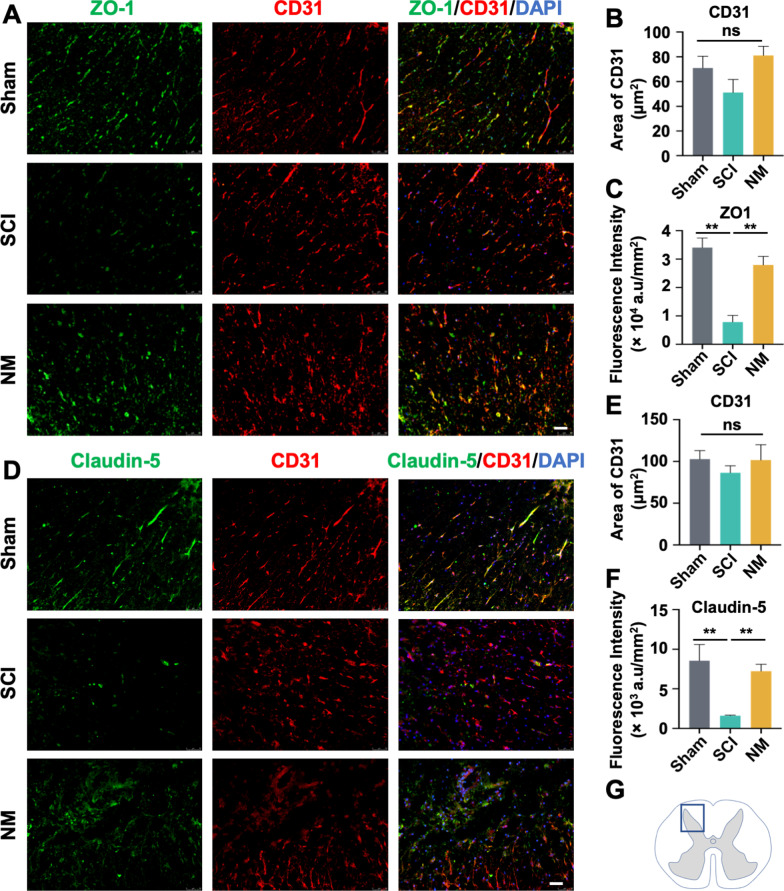
Nafamostat upregulated the tight junction proteins expression after SCI. **A** Immunofluorescence image of CD31 (red) and ZO-1 (green) expression in posterior horn of spinal cord at 3 dpi. Scale bar = 50 μm. **B**, **C** Quantitative analysis of the area (μm^2^) of CD31^+^ cells in the images and the fluorescence intensity of ZO-1. **D** Immunofluorescence image of CD31 (red) and Claudin-5 (green) expression in posterior horn of spinal cord at 3 dpi. Scale bar = 50 μm. **E**, **F** Quantitative analysis of the area (μm^2^) of CD31^+^ cells in the images and the fluorescence intensity of Claudin-5. **G** Illustration describing the image acquisition area in this figure. Fluorescent photos were acquired at the posterior horn of the spinal cord. Cell nuclei were stained with DAPI (blue). All data represent mean ± SEM. ^ns^*P* > 0.05, *P < 0.05, **P < 0.01, ***P < 0.001 vs. injury (one-way ANOVA with Tukey's post hoc test), *n* = 3

Tight junction proteins (TJPs) interconnect ECs of CNS capillaries to maintain the BSCB [33]. Since we found that increased BSCB permeability could be reversed by nafamostat, we asked whether nafamostat altered the expression of TJPs post-SCI. We chose two representative TJPs, ZO-1 and Claudin-5 (Fig. 7A, D). We first investigated the vessel intensity by CD31 after SCI, finding no significant difference between Sham, SCI, and nafamostat group (Fig. 7B, E). The coverage of ZO-1 and Claudin-5 in the epicenter of the spinal cord decreased significantly after SCI, suggesting that many spinal vessels have no barrier function post-SCI. This decrease was reversed by nafamostat treatment at 3 dpi (Fig. 7C, F). These results indicate that nafamostat treatment can prevent BSCB permeability by upregulating the expression of ZO-1 and Claudin-5.

### Nafamostat inhibited macrophage and neutrophil infiltration post-SCI

**Fig. 8:**
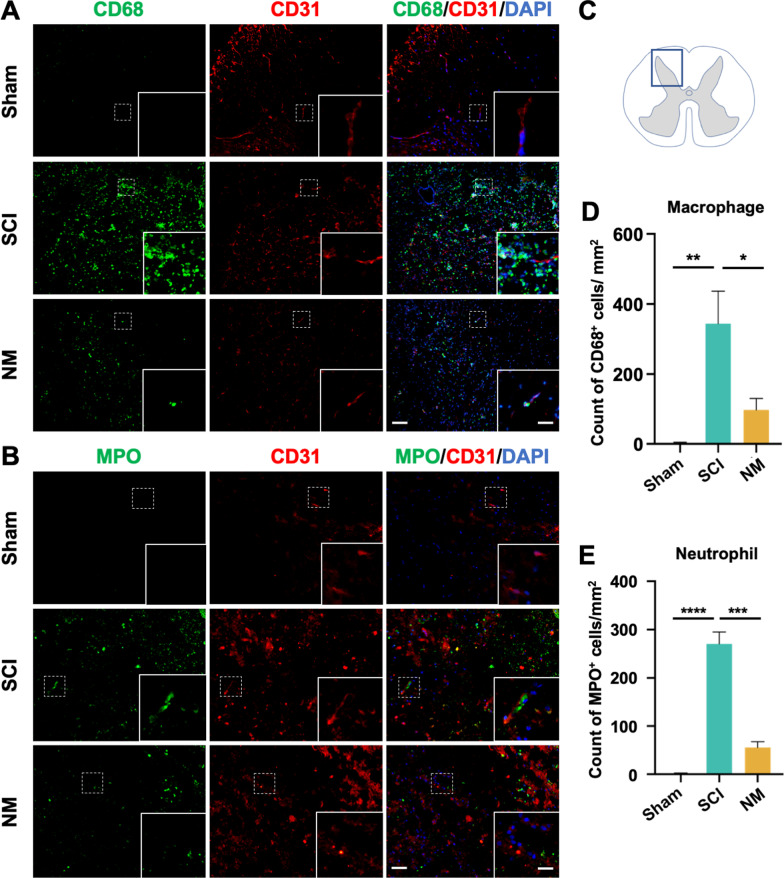
Nafamostat inhibited macrophage and neutrophil infiltration after SCI. **A** Immunofluorescence image of CD31 (red) and CD68 (green) in posterior horn of spinal cord at 3 dpi. Cell nuclei were stained with DAPI (blue). Scale bar = 50 μm. **B** Immunofluorescence image of CD31 (red) and MPO (green) in posterior horn of spinal cord at 3 dpi. Cell nuclei were stained with DAPI (blue). Scale bar = 50 μm. **C** Illustration describing the image acquisition area in this figure. Fluorescent photos were acquired at the posterior horn of the spinal cord. **D** Quantitative analysis of the amount of CD68^+^ cells in posterior horn of spinal cord at 3 dpi. All data represent mean ± SEM. *P < 0.05 and **P < 0.01 vs. injury (one-way ANOVA with Tukey's post hoc test, *n* = 3). **E** Quantitative analysis of the amount of MPO.^+^ cells in posterior horn of spinal cord at 3 dpi. All data represent mean ± SEM. ***P < 0.001, ****P < 0.0001 vs injury (one-way ANOVA with Tukey's post hoc test, *n* = 3)

The inflammatory cascade in acute SCI is mainly derived from peripheral inflammatory cells [34]. The infiltration of peripheral inflammatory cells after injury is attributed to the destruction of BSCB. These inflammatory cells dramatically alter the microenvironment during acute SCI and negatively impact long-term neurological outcome. Since we demonstrated a protective effect of nafamostat on the BSCB, we measured the impact of nafamostat treatment on the infiltration of peripheral macrophage and neutrophil at 3 dpi (Fig. 8A, B). We found a significant decrease in the number of infiltrating CD68^+^ macrophage and MPO^+^ neutrophil in the spinal cord parenchyma of nafamostat group compared with SCI group (Fig. 8D, E). These results suggested that nafamostat could prevent the infiltration of peripheral macrophage and neutrophil into parenchyma to some extent after reducing the permeability of BSCB.

## Discussion

Nafamostat is an FDA-approved drug to treat pancreatitis and is being explored as a treatment for other diseases, such as COVID-19 [35]. We have previously reported that nafamostat has a beneficial effect on recovery from SCI [9, 36]. However, its administration time window and specific mechanism were unclear. In this study, we successfully proved the effective administration time window of nafamostat after SCI and its medication-related safety. Mechanistically, nafamostat repaired SCI through inhibiting the expression and activity of thrombin, and protected ECs from the activation of PAR1 on its surface, then prevented neutrophil and macrophage infiltration and reduced MMP9 release, thereby preventing TJPs degradation, and protected BSCB to repair SCI.

MP is currently the only FDA-approved drug for acute SCI treatment [37]. Although several clinical trials supported that MP should be used within 8 h post-injury, and the duration should not exceed 24 h, there have been many criticisms of MP in recent years because of increasing incidence of complications [3]. Here, we selected nafamostat as a candidate to overcome the issue with MP. Our current study demonstrates that nafamostat is a safe and effective, and has a broader time-window drug for acute SCI repair. In this study, 7 different initial FATs were used to evaluate the optimal administration time window for nafamostat to repair SCI, we observed that administration between 0 h and 12 h after SCI could effectively promote the recovery of long-term locomotor and nerve conduction function. Since nafamostat has an anticoagulant effect, we also tested peripheral blood coagulation function and proved the safety of the administration starting at 2 h after SCI [9]. In terms of overall safety and effectiveness, we determined 2 h to 12 h to be the optimal time window for administration. In conclusion, compared with MP, the advantages of nafamostat for early intervention are obvious, including wider administration time window and lower side effects, which is more suitable for clinical application. Additionally, Liao H et al. have demonstrated the benefit of nafamostat treatment following acute cerebral ischemia [38] via BBB protection, anti-inflammation, and axonal regeneration. Nafamostat can also inhibit thrombin and MMP9 expression to protect brain injury at the early stage of subarachnoid hemorrhage [39].

Here, we also used argatroban, a direct inhibitor of thrombin, as a positive control to prove the decisive role of thrombin in SCI. First, results showed that argatroban and nafamostat had a similar effective time window, which matched the temporal expression pattern of thrombin after SCI. Therefore, we considered that the main mechanism of nafamostat benefit following SCI is thrombin inhibition. Behavioral evaluations showed that argatroban was slightly less effective than nafamostat in SCI repair. As nafamostat is a more broad-spectrum serine protease inhibitor than argatroban, we speculated that although thrombin is the most important factor of serine protease affecting SCI repair, the role of other serine protease systems should not be ignored. Evidence suggests that serine proteases also play an indispensable role in CNS development and disease. Representative serine proteinase, kallikrein (KLK), part of complements (C1r, C1s) and uPA/tPA, have demonstrated involvement in SCI pathological progress [16, 40, 41]. Li Y et al. suggested that regulation of the entire serine protease system can effectively improve scar-free repair in mice with SCI [42]. Therefore, we consider that it is necessary to further explore the mechanism of the role of serine protease system in SCI. Furthermore, various anticoagulants (dabigatran, rivaroxaban, warfarin) are used for anticoagulation in the treatment of SCI, and some serine protease inhibitors (ulinastatin, neuroserpin, pigment epithelium-derived factor) have been shown to be neuroprotective [42–44], our study may provide guidance of administration time window selection for their future clinical application in SCI treatment.

Thrombin is the main trypsin-like serine protease involved in acute SCI pathological progression. Physiologically, neural and glial cells in spinal parenchyma express a low level of endogenous thrombin, which plays a neurodevelopmental and neuroprotective role. However, after SCI, numerous circulation-derived thrombin migrates into the spinal parenchyma following the destruction of BSCB under traumatic stimulation, leading to devastating damage to the nervous system. MMP9 is upregulated during ischemic stroke [45], intracerebral hemorrhage (ICH) [46], and SCI [47], and is highly relevant to thrombin enrichment in spinal parenchyma. Studies have shown that thrombin and MMP9 are very important in the pathogenesis of neurovascular diseases with BBB dysfunction and have a synergistic effect in ICH to aggravate damage [48, 49]. Our study showed that the temporal distribution pattern of thrombin, PAR1 and MMP9 after SCI is similar, and the peak value appears at about 12 h after injury. At the same time, thrombin activity also peaks at 12 h after injury, which is consistent with its expression level. Inhibiting their expression and activity before the peak will likely play an effective role in hindering the development of secondary SCI. Unexpectedly, thrombin activation slightly increased at 7 dpi compared with 3 dpi. We suspect that the increase of thrombin during the subacute stage may have a protective effect on neural injury, on account of evidence shown its neural protective effect with low concentration [50]. Hence, the strange thrombin activation upregulation of thrombin activation at the subacute stage after SCI remains to be explored. In administration time window research, we chose 8–12 h after SCI was the optimal administration time window, which happened to be the peak of thrombin and MMP9. Accordingly, we further proved the rationality of the administration time window on the molecular level. MMP9 expression was increased dramatically through thrombin-activated PAR1 after SCI, further aggravating TJPs degradation, and exacerbated the destruction of BSCB and inflammatory cells in peripheral blood infiltrated into the injured spinal cord [51].

BSCB is a physiological barrier between blood and spinal cord parenchyma, composed of ECs, astrocyte ends, pericyte, as well as basal layer and tight/adhesive junction proteins [52]. The BSCB breakdown after SCI accelerates the exchange of substances between the spinal cord microenvironment and the circulating blood, resulting in a microenvironmental imbalance [34]. Chemokines attract amount of inflammatory cells and pro-inflammatory factors in the serum to the injured spinal cord, leading to an inflammatory cascade [53]. We found that ECs express thrombin receptor PAR1. Moreover, large amount of thrombin accumulated around CD31^+^ vascular ECs, and the administration of nafamostat reduced the expression and activity of thrombin. Meanwhile, MMP9 expression around the vascular ECs decreased significantly after nafamostat intervention, indicating that the activation of PAR1 is a critical factor leading to the loss of TJPs and the BSCB breakdown. We believe that the dramatic increase in thrombin concentration after SCI activates PAR1 and upregulation of MMP9 expression, which induced the destruction of the BSCB, while nafamostat protects the BSCB by inhibiting thrombin activity, thus regulating the imbalance of spinal cord microenvironment after SCI.

Here, we proved that there was no significant difference in BSCB permeability between the groups 7 days after SCI regardless of treatment by EB staining, indicating that BSCB spontaneously recovers during subacute SCI. However, most of the secondary pathological processes after SCI occurred in the acute phase (0–3 dpi) [54], and the protective effect of the BSCB during the acute phase is beneficial for long-term recovery. In this study, 2–12 h administration can effectively protect BSCB from breakdown through inhibiting thrombin and MMP9. Administration of nafamostat at 0 h after injury may promote local bleeding in the injury epicenter to a certain extent, resulting in a slightly worse degree of BSCB breakdown than other treatment groups at 1 day after injury, which once again verified the rationality of the administration time window.

This study still has some limitations. Firstly, we mainly focused on the use of nafamostat in the acute phase of SCI, protecting the BSCB. Observing the role of nafamostat in axonal regeneration after SCI is also worthy of exploration. In this study, we focused on preclinical translation and acute-phase effect, so we used contusion SCI model. Future studies should explore axonal regeneration after SCI of nafamostat treatment in a full transection model or lateral hemisection model, which are usually used for axonal regeneration study in SCI [55]. Secondly, we used young female adult (8-week age) rats for the traumatic SCI preclinical test. Although female rats are used in most pre-clinical research in SCI [56], the gender and age-related difference in the treatment effect of nafamostat need to be further studied. According to epidemiology of traumatic SCI either in US or in China, the incidence rate in males is four or five times higher than female [57, 58], and there is a difference in the recovery between sex in both rat models and human beings in clinic [59]. There is emerging evidence that sex and age are important variables that affect SCI recovery, including on the acute inflammatory response [60]. Therefore, to further push the translation of nafamostat on SCI, more preclinical experiments should be conducted.

In our previous study, the result of inflammation ChIP sequencing showed that nafamostat could reduce many chemokines expression in local site of SCI [36]. Here, we also proved that administration of nafamostat after SCI reduced neutrophil and macrophage infiltration significantly, which we believed that the protection of BSCB was a considerable mechanism of the anti-neuroinflammation effect induced by nafamostat.

## Conclusions

**Fig. 9:**
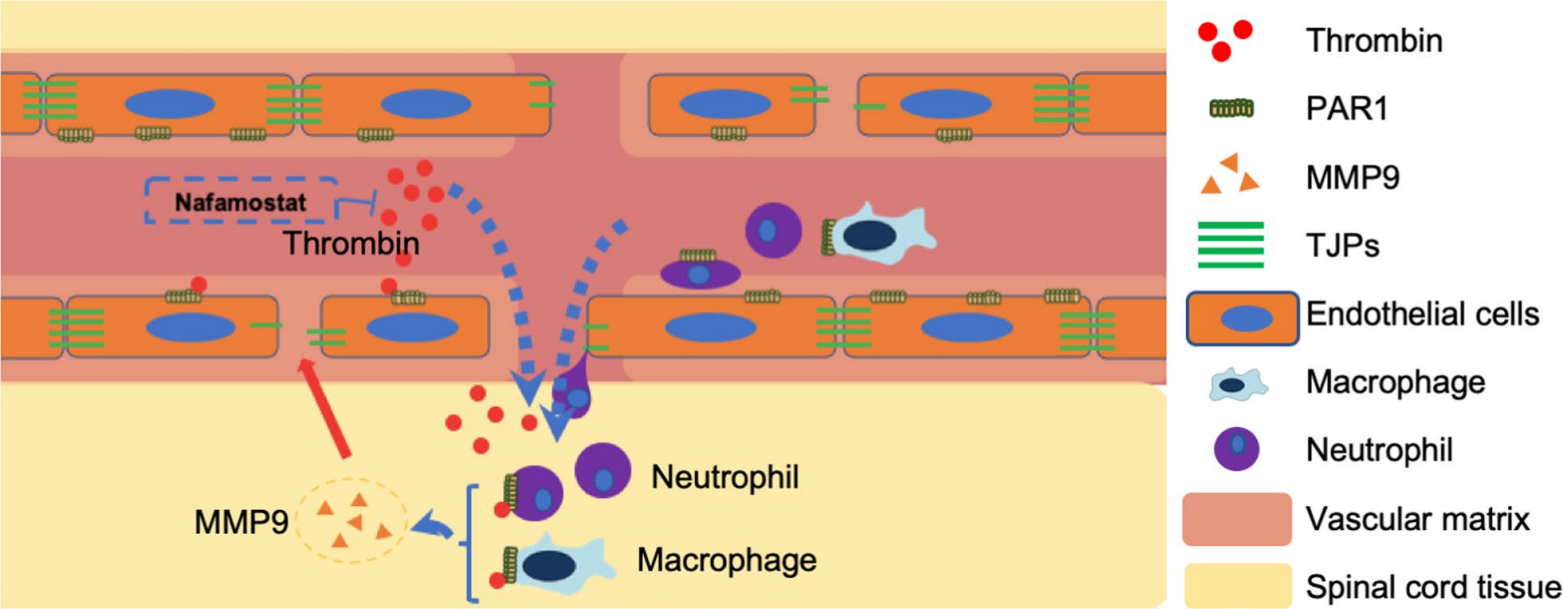
Schematic of the mechanism of nafamostat on SCI. Graphical abstract of mechanism of nafamostat repairing SCI

Our study provides preclinical data of nafamostat optimal administration time window of 2–12 h administration post-injury in SCI for the first time. These results are potentially clinically relevant for acute SCI (Fig. 9). We demonstrated that administration of nafamostat can be effective in inhibiting the thrombin/PAR1/MMP9 axis, which protects ECs and TJPs, reduces infiltration of peripheral immune cells, and improves SCI recovery.

## Supplementary Information

**Additional file 1: Figure S1. **Different administration time points of Argatroban improved functional recovery after SCI. (A). The degree of hindlimb recovery was assessed for 5 w after SCI by BBB score. 7 different first administration time points of argatroban were 0 h, 2 h, 8 h, 12 h, 24 h, 5 dpi. (Data shown as mean ± SEM, two-way ANOVA with Tukey’s post hoc test, *P < 0.05, **P < 0.01, ***P < 0.001 vs. the SCI group, *n* = 3). (B). Comparison of BBB scores of each group at 5 w point post-injury. (Data shown as mean ± SEM, one-way ANOVA with Tukey's post hoc test, *P < 0.05, **P < 0.01, ***P < 0.001 vs. the SCI group, *n* = 6). (C). Representative SEP and MEP waveform of nerve electrophysiology examination of rats in each group at 5 w. (D-G). Quantification of the amplitude and latency of SEP and MEP in each group at 5 w point. (Data shown as mean ± SEM, one-way ANOVA with Tukey's post hoc test, *P < 0.05, **P < 0.01, ***P < 0.001 vs. the SCI group, *n* = 6).

**Additional file 2**: **Figure S2.** Nafamostat administration within an appropriate time window did not affect the coagulation function after SCI. (A, B, C). Representative changes in prothrombin time (PT), prothrombin activity (PTA), prothrombin time international normalized ratio (PT-INR) 2 h after administration in rats of each group at different administration time points. (PT: time for activation of prothrombin to thrombin; PTA: an indicator reflecting thrombin activity; PT-INR: an indicator that reflects the time it takes for blood to clot. Data shown as mean ± SEM, unpaired t-test, *P < 0.05, **P < 0.01, ***P < 0.001 vs. the SCI group, *n* = 3).

**Additional file 3: Figure S3**. Representative co-localized images of thrombin (red) and neuron (NeuN, A), Oligodendrocyte (Olig2, B), astrocytes (GFAP, C), microglia (Iba1, D), pericytes (PDGFR-β, E) and ECs (CD31, F) at 3 dpi. Cell nuclei were stained with DAPI (blue). Scale bar = 50 μm, n = 3.

**Additional file 4: **Figure S4. Leakage of fibrinogen in the spinal cord parenchyma after SCI. (A). Immunofluorescence image of CD31 (red) and Fibrinogen (green) at 3dpi. Cell nuclei were stained with DAPI (blue). Scale bar = 25 μm. Orange arrow: Fibrinogen was confined to the blood vessels; white arrow: Fibrinogen exudated through the blood vessels and infiltrated into the spinal parenchyma. (B). Illustration describing the image acquisition area in this figure. Fluorescence was acquisition at the posterior horn of the spinal cord. (C). Quantitative analysis of the fluorescence intensity of Fibrinogen. (Data shown as mean ± SEM, one-way ANOVA with Tukey's post hoc test, **P < 0.01, ***P < 0.001 vs. the SCI group, n = 3).

## Data Availability

The data obtained from this study are included in the article or the Additional files.

## Abbreviations

NM: Nafamostat mesylate
SCI: Spinal cord injury
PAR1: Protease activated receptor 1
MMP9: Matrix metalloproteinase 9
ECs: Endothelial cells
BSCB: Blood–spinal cord barrier
TJPs: Tight junction proteins
MP: Methylprednisolone
CNS: Central nervous system
BBB: Blood–brain barrier
TBI: Traumatic brain injury
ZO-1: Zonula occludens-1
FAT: First administration time
RI: Regularity index
SEP: Sensory evoked potential
MEP: Motor evoked potential
PT: Prothrombin time
PTA: Prothrombin activity
INR: International normalized ratio
EB: Evans Blue
SEM: Standard error and mean
