# Supplementary figures and images for: Delayed administration of nafamostat mesylate inhibits thrombin-mediated blood–spinal cord barrier breakdown during acute spinal cord injury in rats
Source: J Neuroinflammation. 2022 Jul 16;19:189. doi: 10.1186/s12974-022-02531-w (PMC9287720; doi:10.1186/s12974-022-02531-w)

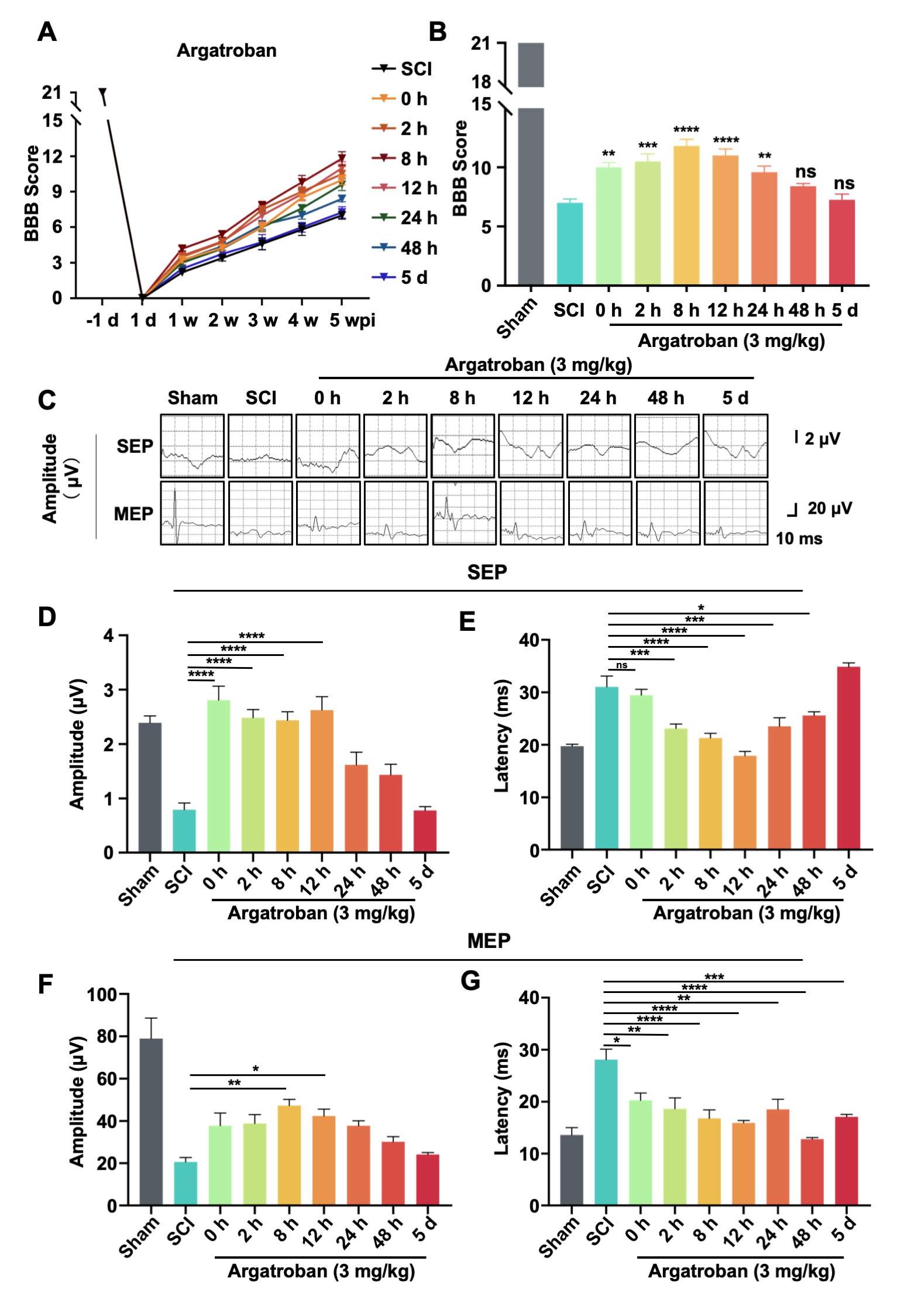

Supplement: Supplementary file 1 — Additional file 1: Figure S1. Different administration time points of Argatroban improved functional recovery after SCI. (A). The degree of hindlimb recovery was assessed for 5 w after SCI by BBB score. 7 different first administration time points of argatroban were 0 h, 2 h, 8 h, 12 h, 24 h, 5 dpi. (Data shown as mean ± SEM, two-way ANOVA with Tukey’s post hoc test, *P < 0.05, **P < 0.01, ***P < 0.001 vs. the SCI group, n = 3). (B). Comparison of BBB scores of each group at 5 w point post-injury. (Data shown as mean ± SEM, one-way ANOVA with Tukey's post hoc test, *P < 0.05, **P < 0.01, ***P < 0.001 vs. the SCI group, n = 6). (C). Representative SEP and MEP waveform of nerve electrophysiology examination of rats in each group at 5 w. (D-G). Quantification of the amplitude and latency of SEP and MEP in each group at 5 w point. (Data shown as mean ± SEM, one-way ANOVA with Tukey's post hoc test, *P < 0.05, **P < 0.01, ***P < 0.001 vs. the SCI group, n = 6). [file 12974_2022_2531_MOESM1_ESM.tiff]

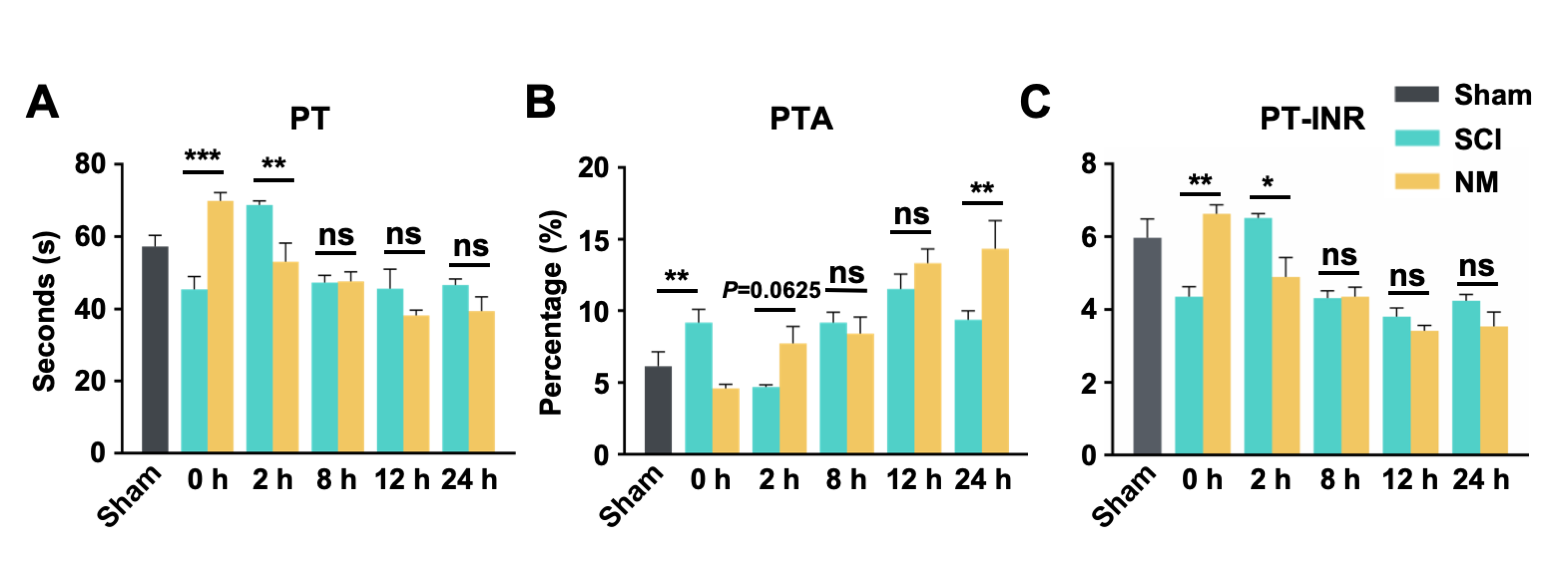

Supplement: Supplementary file 2 — Additional file 2: Figure S2. Nafamostat administration within an appropriate time window did not affect the coagulation function after SCI. (A, B, C). Representative changes in prothrombin time (PT), prothrombin activity (PTA), prothrombin time international normalized ratio (PT-INR) 2 h after administration in rats of each group at different administration time points. (PT: time for activation of prothrombin to thrombin; PTA: an indicator reflecting thrombin activity; PT-INR: an indicator that reflects the time it takes for blood to clot. Data shown as mean ± SEM, unpaired t-test, *P < 0.05, **P < 0.01, ***P < 0.001 vs. the SCI group, n = 3). [file 12974_2022_2531_MOESM2_ESM.tiff]

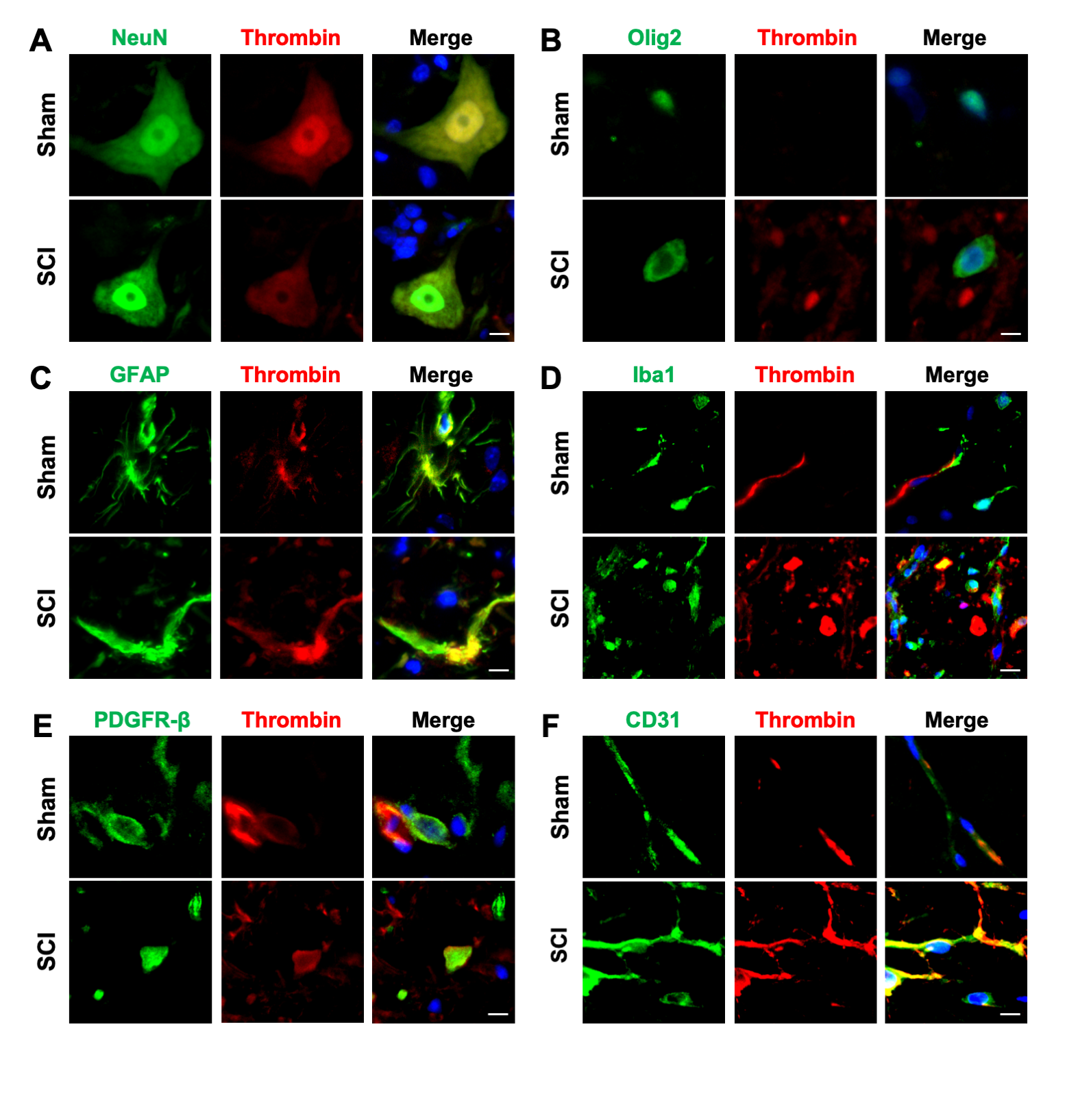

Supplement: Supplementary file 3 — Additional file 3: Figure S3. Representative co-localized images of thrombin (red) and neuron (NeuN, A), Oligodendrocyte (Olig2, B), astrocytes (GFAP, C), microglia (Iba1, D), pericytes (PDGFR-β, E) and ECs (CD31, F) at 3 dpi. Cell nuclei were stained with DAPI (blue). Scale bar = 50 μm, n = 3. [file 12974_2022_2531_MOESM3_ESM.tiff]

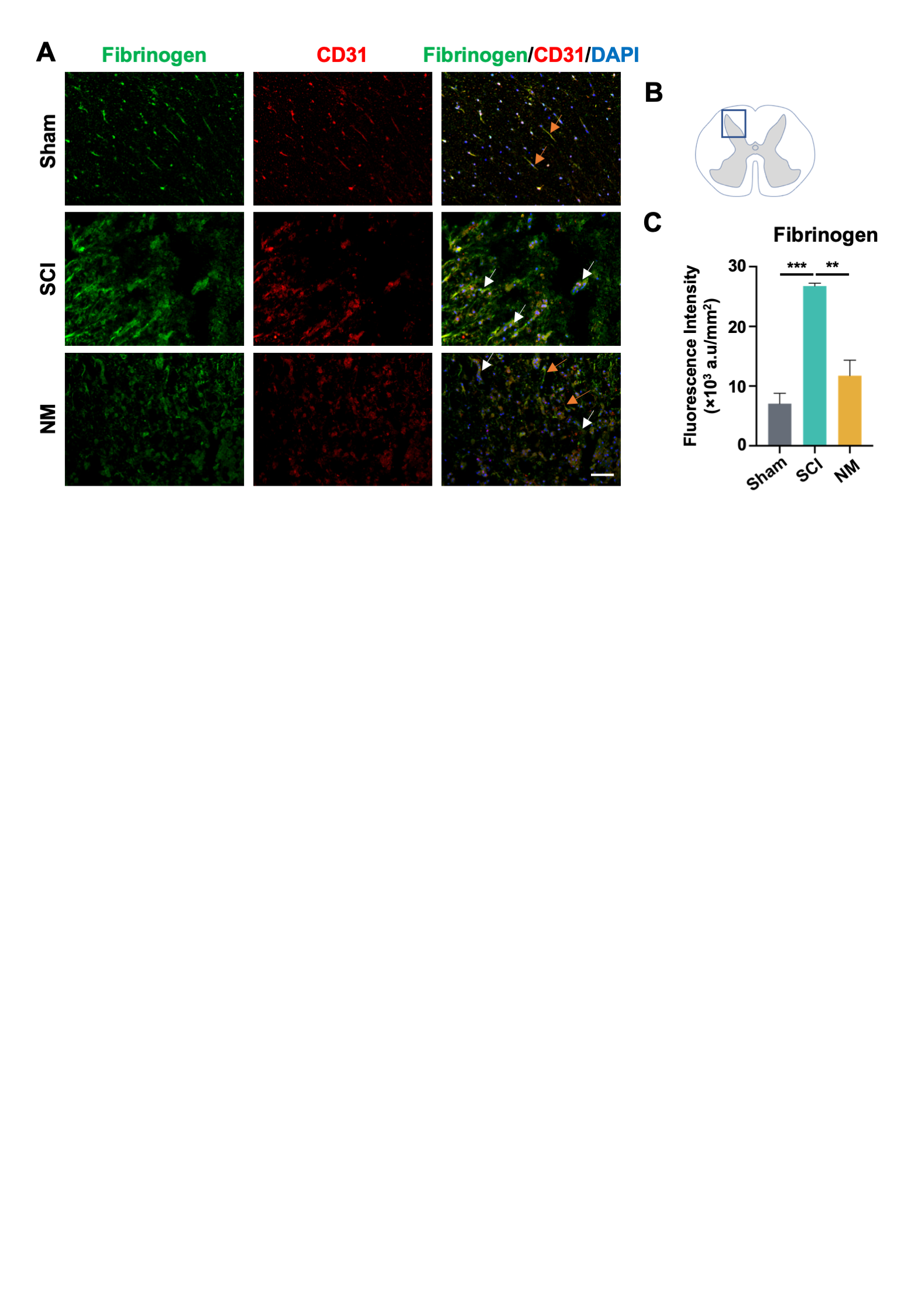

Supplement: Supplementary file 4 — Additional file 4: Figure S4. Leakage of fibrinogen in the spinal cord parenchyma after SCI. (A). Immunofluorescence image of CD31 (red) and Fibrinogen (green) at 3dpi. Cell nuclei were stained with DAPI (blue). Scale bar = 25 μm. Orange arrow: Fibrinogen was confined to the blood vessels; white arrow: Fibrinogen exudated through the blood vessels and infiltrated into the spinal parenchyma. (B). Illustration describing the image acquisition area in this figure. Fluorescence was acquisition at the posterior horn of the spinal cord. (C). Quantitative analysis of the fluorescence intensity of Fibrinogen. (Data shown as mean ± SEM, one-way ANOVA with Tukey's post hoc test, **P < 0.01, ***P < 0.001 vs. the SCI group, n = 3). [file 12974_2022_2531_MOESM4_ESM.tiff]
